# Degree of dyspnoea at admission and discharge in patients with heart failure and respiratory diseases

**DOI:** 10.1186/s12904-017-0208-x

**Published:** 2017-05-22

**Authors:** Lourdes Vicent, Juan Manuel Nuñez Olarte, Luis Puente-Maestu, Alicia Oliva, Juan Carlos López, Andrea Postigo, Irene Martín, Raquel Luna, Francisco Fernández-Avilés, Manuel Martínez-Sellés

**Affiliations:** 1 Department of Cardiology, Hospital General Universitario Gregorio Marañón, Doctor Esquerdo 46, 28007 Madrid, Spain; 2 Department of Palliative Care, Hospital General Universitario Gregorio Marañón, Madrid, Spain; 3 Universidad Complutense, Madrid, Spain; 4 Department of Respiratory Medicine, University Hospital Gregorio Marañón, Madrid, Spain; 5 Universidad Europea, Madrid, Spain

**Keywords:** Dyspnoea, Heart failure, Chronic obstructive pulmonary disease, Morphine, EuroQoL 5d

## Abstract

**Background:**

Dyspnoea is a disabling symptom in patients admitted with heart failure (HF) and respiratory diseases (RD). The main aim of this study is to evaluate its intensity at admission and discharge and the relation with quality of life. We also describe its management, intensity, and evolution in HF and RD.

**Methods:**

In this descriptive, cross-sectional study, we included prospectively all patients admitted with decompensated HF and chronic obstructive pulmonary disease (COPD)/pulmonary fibrosis during 4 months. Surveys quantifying dyspnoea (Numerical Rating Scale 1-10) and quality of life (EuroQoL 5d) were administered at discharge.

**Results:**

A total of 258 patients were included: 190 (73.6%) with HF and 68 (26.4%) with RD (62 COPD and 6 pulmonary fibrosis). Mean age was 74.0±1.2 years, and 157 (60.6%) were men. Dyspnoea before admission was 7.5±0.1. Patients with RD showed greater dyspnoea than those with HF both before admission (8.1±0.2 vs. 7.3±0.2, *p*=0.01) and at discharge (3.2±0.3 vs. 2.0±0.2, *p*=0.0001). They also presented a higher rate of severe dyspnoea (≥5) at discharge (23 [34.3%] vs. 36 [19.1%], *p*=0.02). Opioids were used in 41 (15.9%), mean dose 8.7±0.8 mg Morphine Equivalent Daily Dose. HF patients had worse EuroQoL 5d scores than those with RD, due to mobility problems (118 [62.1%] vs. 28 [41.8%], *p*=0.004), and lower punctuation in Visual Analogue Scale (57.9±1.6 vs. 65.6±1.0, *p*=0.006).

**Conclusions:**

About a quarter of patients admitted with HF or RD persist with severe dyspnoea at discharge. Opioids are probably underused. HF patients have less dyspnoea than patients with RD but present worse quality of life.

**Electronic supplementary material:**

The online version of this article (doi:10.1186/s12904-017-0208-x) contains supplementary material, which is available to authorized users.

## Background

Dyspnoea is one of the most disabling symptoms of heart failure (HF) and respiratory diseases (RD), [1] and the main reason that leads to hospital admission [2]. This symptom surpasses the physical dimension and has a major social and emotional impact [3]. Despite its importance and the well-validated scales available, dyspnoea is not routinely assessed [4, 5]. The way to express breathlessness by patients is variable, and it can be difficult to assess objectively the intensity of dyspnoea [6]. In addition, many patients tend to self-limit the intensity of their physical activity and might not feel incapacitating dyspnoea despite being in poor functional class [7]. An effort to quantify the severity of breathlessness is justified, since it is a factor of poor prognosis and is associated with worse outcomes, higher mortality rates, and longer hospital stay [8]. Due to these reasons dyspnoea has been recently recommended as a main endpoint of clinical trials [4]. However, there are few studies that specifically assess the severity of breathlessness, especially in the cardiovascular field [4, 5]. Most of the studies of dyspnoea that have been carried out in HF have been derived from post hoc analysis of large randomized trials [2, 9]. There are few studies designed specifically to measure the intensity and impact of breathlessness on quality of life and the potential improvement that can be achieved after hospital admission [10]. Some patients remain symptomatic at hospital discharge, and would be eligible to receive additional treatment over standard therapies. Some patient’s and treatment characteristics may be associated with a higher probability of presenting severe dyspnea. The identification of these characteristics could be useful to prescribe medications with proven benefit, such as opioids.

The main aim of this study is to evaluate the intensity of dyspnoea in patients hospitalised for HF and RD (chronic obstructive pulmonary disease [COPD] and pulmonary fibrosis) at admission and discharge, and its relation with quality of life. We also describe dyspnoea management (especially taking into account the use of opioids) and compare its intensity and evolution in the two groups. Additionally, we aim to ascertain the factors associated with the persistence of a severe intensity of dyspnea at hospital discharge.

## Methods

We performed a descriptive study of dyspnoea performed in Cardiology and Respiratory Medicine Departments using a cross-sectional design in order to assess the magnitude of the problem of dyspnea.

“Hospital without dyspnoea” is a multidisciplinary initiative started in 2014 that aims to reduce the prevalence of dyspnoea in hospitalised patients in our centre with a multidisciplinary approach that includes the departments of Palliative Care, Cardiology, and Respiratory Medicine [11]. We have designed a multiphase quasi-experimental study with a teaching intervention addressed to healthcare professionals. The first phase of this protocol is the present one.

### Study population, endpoints and data collection

We prospectively included all adult patients admitted for HF, and RD exacerbations in our centre from April to September 2016. Exclusion criteria were inhospital death, severe cognitive impairment and not providing consent to participate in the study. All patients were being optimally treated according to standard disease management guidelines. We collected demographic variables, previous medical history (HF, valvular heart disease, ischemic heart disease, respiratory diseases, peripheral arteriopathy, stroke with sequels, presence of cognitive impairment and previous hospital admissions), variables related with treatment before admission (oxygen and continuous positive airway pressure use) and hospitalization variables such as the cause of exacerbation, intensive care unit admission, invasive or non-invasive mechanical ventilation, and diuretics, opioid, benzodiazepines or antidepressant drugs administration.

Medical history data were extracted from previous medical records. Heart failure was defined according to guidelines recommendations [12]. Patients were classified in two groups “preserved and reduced ejection fraction”, with a cut-off point of 45%. All patients with heart failure and preserved ejection fraction had diastolic dysfunction defined as the presence of a dilated left atrium (left atrium volume index >34 mL/m2), increased left ventricular mass or functional abnormalities, such as an impaired relaxation pattern in echocardiography [12]. COPD was defined as the presence of a post-bronchodilator forced expiratory volume/forced vital capacity relationship less than 0.70. Severity was graded according to post-bronchodilator predicted forced expiratory volume in three groups (mild ≥80%, moderate 50-80% and severe/very severe <50%) [13]. Pulmonary fibrosis [14, 15] was diagnosed if a reticular pattern was present in imaging tests and gas exchange at rest and during exercise was reduced (predicted single-breath carbon monoxide transference ≤60% and desaturation during 6 minutes walk test).

All patients who were admitted for respiratory disease or heart failure were invited to participate in the registry.

Informed consent was obtained from each patient and the study protocol conforms to the ethical guidelines of the 1975 Declaration of Helsinki as reflected in a priori approval by the institution's human research committee.

### Dyspnoea measurement

In order to quantify the intensity of dyspnoea, patients were asked to answer some questions from a questionnaire. Five physicians (JCL, RL, IM, AP, AO) recruited the patients and administered the questionnaires the day of hospital discharge. Patients were asked to select from a 0-10 numerical rating scale [16] the grade of dyspnea they felt at the moment of admission, the worse breathlessness during hospitalization, and at discharge. Severe dyspnoea at discharge was defined as the persistence of a dyspnoea intensity ≥ 5 points.

### Additional assessments

The assessment also included New York Heart Association functional class, pain evaluation on a 0 to 10 scale (disabling degree was considered to be present if ≥ 5), methods used to relieve breathlessness and communication with primary care doctor about dyspnoea before admission.

### Quality of life measurement

We used the EuroQoL 5d [17] quality of life classification. The questionnaire is detailed in Additional file 1: Appendix 1.

The EuroQuol 5d is an instrument for measuring the quality of life related to health. The patient values his health in severity levels in five dimensions (mobility, personal care, daily activities, pain, anxiety) and also on a visual analog scale. Each of the 5 dimensions has 3 levels of potential severity: no problems, some problems or moderate problems and severe problems. The analog scale assessment consists of a millimetre line from 0 (worst imaginable health state) to 100 (best imaginable health state). A third element is the global index values obtained for each health status generated by the instrument, which can range from the value 1 (the better health posible) and 0 (death). There are also negative values that are rated as worse than death [18–20].

Depression was valued according to the degree of limitation noted by patients in the “anxiety/depression” dimension of EuroQoL 5d. In this questionnaire, the emotional state is measured in three levels of severity, from 1 (“no problems”) to 3 (“extreme problems”). Patients with 2 or 3 points in this dimension have a substantial mood disorder.

### Utilization of opioids

Morphine was considered as the opioid of reference and is the only opioid currently indicated for dyspnoea relief. Other derivatives can be considered as an alternative to patients intolerant to morphine [21, 22]. We also recorded the number of patients receiving other opioid derives for chronic pain relief. In all cases opioids were used specifically to relieve dyspnoea. In order to quantify the effect of the administered dose in patients receiving treatment with other opioids and assuming the oral route of opioid administration as a point of reference for comparisons, the Morphine Equivalent Daily Dose (MEDD) was calculated. This equivalence was extracted from dose tables that specify dosage equianalgesic efficacy [23].

### Statistical analysis

Continuous variables are presented as mean with standard deviation, 95% confidence intervals or median with interquartile range. Categorical data are showed as frequencies and percentages. Comparisons of continuous variables were made using the *t* test for normally distribution data or Wilcoxon rank sum in nonparametric data. Binary variables were contrasted using the χ^2^ test and Fisher’s exact test. These statistical tests were used to make group comparisons in Tables 1, 2, 3, 4 and 5. Independent predictors of mortality were determined using a logistic regression model. The modelling process involved forward and backward stepwise methods with a threshold for exit set at *P* higher than 0.10 and for enter at *P* lower than 0.10. All statistical analysis were two-tailed. Statistical analysis was performed with the statistical software of STATA (14.0 version).

**Table 1:** Baseline characteristics and comorbidities

	Total *N*=258	Heart Failure *N*=190	Respiratory Disease *N*=68	*p*
Age, years	74.0±1.2	73.5±1.4	75.1±1.9	0.53
Male Sex	157 (60.9)	104 (54.7)	53 (78.0)	0.0001
Previous history of heart disease	170 (65.9)	149 (78.4)	21 (30.8)	0.0001
Sinus Rhythm	138 (53.5)	78 (41.1)	60 (88.2)	0.001
Ischemic Heart Disease	106 (41.1)	91 (47.9)	15 (22.1)	0.001
Valvular Heart Disease	87 (33.7)	73 (38.4)	14 (20.6)	0.02
Left ventricular ejection fraction <45%	102 (39.5)	99 (52.1)	3 (4.4)	0.0001
Unknown left ventricular ejection fraction	18 (7.0)	0	18 (26.4)	0.0001
Known Right ventricle dysfunction	62 (24.0)	59 (31.1)	3 (4.4)	0.001
Known Pulmonary Hypertension	104 (40.3)	82 (43.2)	12 (17.6)	0.001
Obstructive Sleep Apnea Syndrome	30 (11.6)	17 (8.9)	13 (19.1)	0.03
Peripheral Arterial Disease	31 (12.0)	24 (12.6)	7 (10.3)	0.67
Stroke	16 (6.2)	15 (7.9)	1 (1.5%)	0.03
Cognitive impairment	8 (3.1)	5 (2.6)	3 (4.4%)	0.48
Previous hospital admissions				0.0001
Heart Failure	102 (39.5)	101 (53.2)	1 (1.5)	
COPD or Pulmonary Fibrosis	49 (19.0)	1 (0.5)	48 (70.6)	
Domiciliary oxygen	32 (12.4)	5 (2.6)	27 (39.7)	0.0001
Continuous Positive Airway Pressure at home	24 (9.3)	13 (6.8)	11 (16.2)	0.03
Length of stay	8.7±0.5	9.3±0.6	7.3±0.4	0.06
Living alone	64 (24.8)	46 (24.2)	18 (26.5)	0.87
Lives with family	190 (73.6)	142 (74.7)	48 (70.6)	
Living in residency	3 (1.2)	2 (1.1)	1 (1.5)	

Data are shown as number of patients and percentages (mean±standard deviation for age). Categorical variables are contrasted using the χ^2^ test and Fisher’s exact test and comparison for age is made using the *t* test

**Table 2:** Treatment administered during admission and methods recognized by the patients themselves for dyspnea relief prior to hospitalization

	Total *N*=258	Heart Failure *N*=190	Respiratory Disease *N*=68	*p*
Location and treatment administered during admission				
Intensive care unit admission	71 (27.5)	62 (32.6)	9 (13.2)	0.002
Non-invasive mechanical ventilation	48 (18.6)	31 (16.3)	17 (25.0)	0.11
Diuretics	216 (83.7)	187 (98.4)	29 (42.6)	0.0001
Bronchodilators	85 (33.0)	32 (16.8)	53 (77.9)	0.0001
Benzodiazepines	138 (53.4)	112 (58.9)	26 (38.2)	0.004
Opioids	41 (15.9)	34 (17.9)	7 (10.3)	0.17
MEDD (mg)	8.7±0.8	8.0±0.6	12.4±3.3	0.03
Antidepressants	34 (13.2)	24 (12.6)	10 (14.9)	0.67
Methods used for dyspnoea relief prior to hospitalization				
Sleeping with high headboard	140 (54.3)	115 (60.5)	25 (36.8)	0.001
Fan	16 (6.2)	15 (7.9)	1 (1.5)	0.03
Oxygen	46 (17.8)	15 (7.9)	31 (45.6)	0.0001
Continuous Positive Airway Pressure	24 (9.3)	11 (5.8)	13 (19.1)	0.003
Inhalers	91 (35.3)	37 (19.5)	54 (79.4)	0.0001
None	58 (22.5)	51 (26.8)	7 (10.3)	0.003
Communication with GP regarding dyspnoea	153 (59.3)	98 (51.6)	54 (79.4)	0.0001
Seeking for medical attention during admission due to acute dyspnoea	96 (37.2)	73 (38.4)	23 (33.8)	0.53
Thinks that treatment received could be better	91 (35.3)	65 (34.2)	26 (38.2)	0.57

*GP* General practitioner, *MEDD*, Morphine Equivalent Daily Dosage

Data are shown as number of patients and (mean±standard deviation for Mean Morphine Equivalent Daily Dosage [MEDD]). Categorical variables are contrasted using the χ^2^ test and Fisher’s exact test and comparison for MEDD is made using the *t* test

**Table 3:** Univariate analysis of severe dyspnoea-related factors at hospital discharge

	Dyspnoea <5 points *N*=199	Dyspnoea ≥5 points *N*=59	*p*
Age >70 years	44 (22.1)	18 (30.5)	0.221
Female Sex	86 (43.2)	15 (25.4)	0.009
Respiratory Disease	44 (22.1)	23 (39.0)	0.017
History of heart disease	129 (64.8)	40 (67.8)	0.876
Sinus rhythm	103 (51.8)	34 (57.6)	0.552
Preserved ejection fraction	86 (43.2)	17 (28.8)	0.08
Severe COPD	26 (13.1)	16 (27.1)	0.016
Previous admission for exacerbation	103 (51.8)	47 (79.7)	<0.0001
Domiciliary oxygen therapy	17 (8.5)	16 (27.1)	0.001
Admission in the intensive care unit	56 (28.1)	14 (23.7)	0.21
Non-invasive mechanical ventilation	33 (16.6)	14 (23.7)	0.38
Opioid treatment	32 (16.1)	20 (33.9)	0.003
Mobility problems	100 (50.3)	47 (79.7)	<0.0001
Depression	73 (36.7)	41 (70.5)	<0.0001

*COPD* Chronic Obstructive Pulmonary Disease

Data are shown as number of patients and percentages. All variables are contrasted using the χ^2^ test and Fisher’s exact test

**Table 4:** Independent predictors of severe dyspnoea (≥5 points) at hospital discharge by logistic regression model

Variable	OR (95% CI)	*P*
Age	1.1 (1.02-1-2)	0.004
Female Sex	2.2 (1.2-2.3)	0.005
Depression	3.9 (2.1-7.2)	0.001
Non admitted in an intensive care unit	3 (1.1–8.3)	0.05
Respiratory Disease	1.2 (1.1-1.4)	0.001

*N*=258; *CI* Confidence Interval, *OR* Odds Ratio

**Table 5:** Patients presenting limitations in EuroQoL 5d

	Total *N*=258	Heart Failure *N*=190	Respiratory Disease *N*=68	*p*
Mobility	146 (56.6)	118 (62.1)	28 (41.2)	0.004
Self-care	96 (37.2)	73 (38.4)	23 (33.8)	0.55
Usual activities	160 (62.0)	119 (62.6)	41 (60.3)	0.88
Pain/discomfort	105 (40.7)	82 (43.2)	23 (33.8)	0.25
Anxiety/depression	114 (44.2)	86 (45.3)	28 (41.2)	0.62
Visual analogic scale	59.9±1.3	57.9±1.6	65.6±1.0	0.006
EuroQoL Index	0.63±0.02	0.60±0.03	0.73±0.04	0.01

Data are shown as number of patients and mean±standard deviation for Visual analogic scale and EuroQoL Index. Categorical variables are contrasted using the χ^2^ test and Fisher’s exact test. Comparisons for Visual analogic scale and EuroQoL Index are made using the *t* test

## Results

During the study period, 268 patients were screened to participate. Finally 258 patients (157 men, 60.6%) were included as six patients were excluded due to moderate or severe cognitive impairment and four refused to enter the registry. The number of unanswered questions was very low: only eight patients had significant difficulties to answer the survey questions, and all of them left blank the Numerical Rating Scale. A total of 190 (73.6%) were admitted in the Cardiology Department due to HF decompensation and 68 (26.4%) in the Respiratory Medicine Department due to RD (62 COPD and six pulmonary fibrosis). Mean age was 74.0±1.2 years, and 36 (14.0%) were octogenarians. Baseline characteristics, medical history, and length of stay are shown in Table 1. Left ventricular dysfunction, ischemic or valvular heart disease, rhythm disturbances, right ventricular dysfunction, and pulmonary hypertension were more usual in patients suffering from HF, than in those with RD. The opposite happened with obstructive sleep apnoea and domiciliary oxygen therapy. We found a trend towards longer length of stay in patients who presented with severe dyspnoea at admission (11.4±1.8 vs. 8.4±0.5 days in those without severe dyspnoea at admission, *p*=0.06).

### Dyspnoea management

Treatments administered during admission are detailed in Table 2. Patients suffering from HF were more often admitted to intensive care units and received diuretics and benzodiazepines more frequently than patients with RD. Eight patients received tramadol (3.1%) and one patient was treated with codeine (0.4%).

In patients treated with opioids, 15 (36.6%) received continuous treatment and 26 (63.4%) only intermittent administration for dyspnoea crisis management. The mean opioid dose was 8.7±0.8 mg MEDD, with higher doses in patients with RD (12.4±3.3 mg *vs*. 8.0±0.6 mg in HF, *p*=0.03). Patients admitted to Critical Care Units were more commonly treated with opioids (17 [23.9%] vs. 24 [12.8%] in those admitted directly to general wards, *p*=0.04). Opioid prescription was also more frequent in patients that persisted with severe dyspnoea at discharge (16 [27.1%] vs. 25 [12.7%] in patients with milder dyspnoea at discharge, *p*=0.01), as was continuous opioid administration during admission (9 [56.3%] vs. 6 [24.0], respectively, *p*=0.04). The dosage of opioids was higher in patients with a continuous treatment (11.3±1.8 mg *vs*. 7.2±0.5 mg in those without, *p*=0.01), but the amount of drug administered was not related to the degree of dyspnoea at discharge (severe dyspnoea 9.6±1.7 mg vs. 8.3±0.7 mg in milder dyspnoea, *p* = 0.41). At hospital discharge, only 16 patients with severe dyspnoea (27.1%) received specific treatment with opioids for dyspnoea relief.

Table 2 shows the methods used for dyspnoea relief prior to hospitalization, and former communication with the primary care physician regarding breathlessness. Patients admitted for RD had previously consulted with their general practitioner for this reason more frequently than those with HF. The most frequent method used for the relief of dyspnoea in HF patients was sleeping with more than one pillow or the head of the bed elevated. On the other hand, continuous positive airway pressure, inhalers, and supplemental oxygen were more common in RD patients.

### Degree of perceived dyspnoea and quality of life impairment

**Fig. 1:**
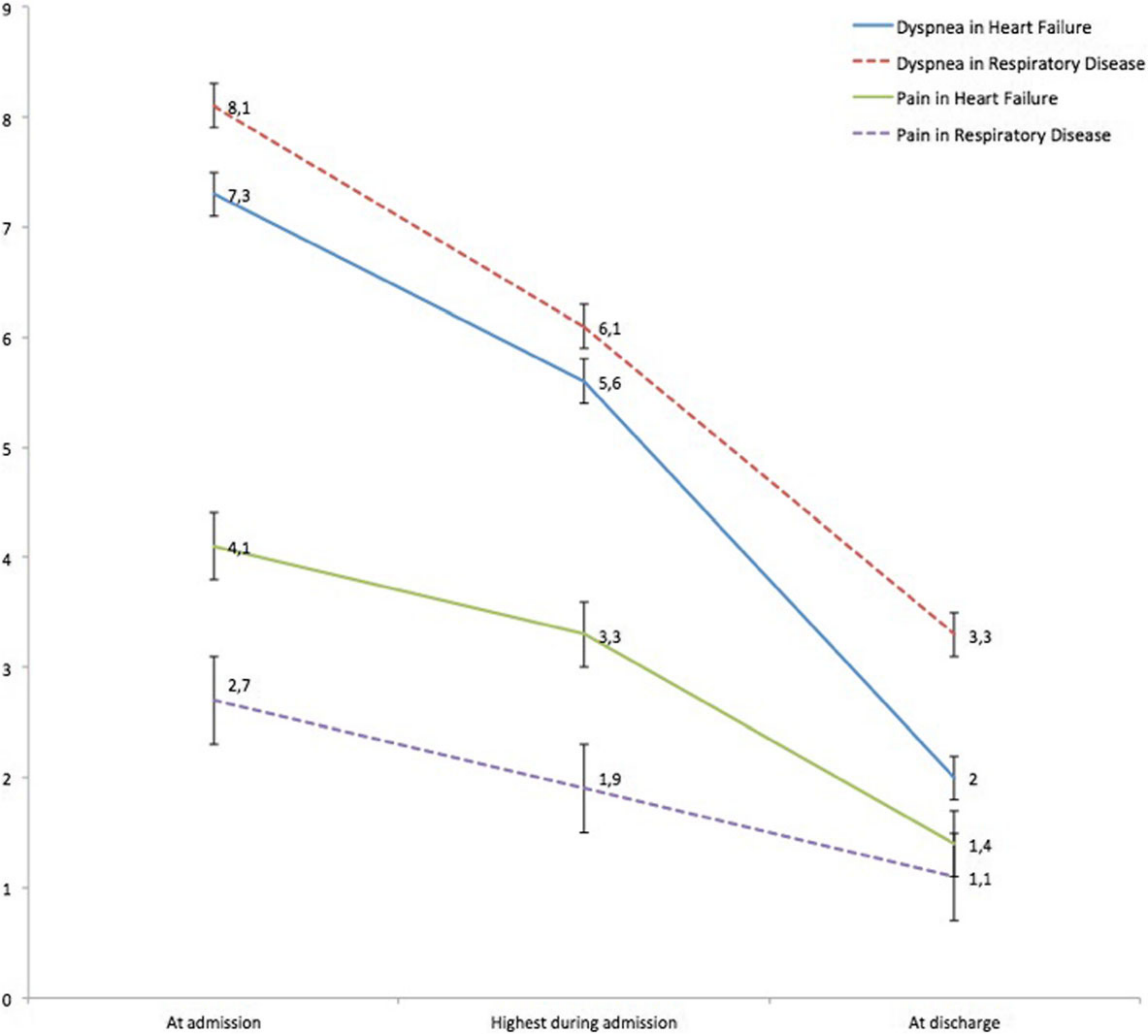
Progression of dyspnoea and pain during hospital admission. The figure shows the evolution of dyspnoea and pain during admission in patients with heart failure (*continuous line*) and respiratory diseases (*dashed line*). The data represent the mean value according to the Numerical Rating Scale in three moments: at admission, the maximum value perceived during admission and hospital discharge. Error bars represent the standard deviation. “Highest during admission” refers to the highest value of dyspnoea or pain perceived during the course of hospitalization after excluding the symptoms at the time of admission

Dyspnoea severity during hospital admission is shown in Fig. 1. Patients with RD reported higher intensity of breathlessness than those with HF. The degree of dyspnoea improved during hospitalization with a similar reduction in both groups (5.2±0.2 points in HF vs. 4.9±0.3 points in RD, *p*=0.41). Fifty-nine patients (23.1%) still presented severe dyspnoea (>5 points) at discharge (36 [19.1%] in HF vs. 23 [34.3%] in RD, *p*=0.02). Patients admitted to intensive care units presented lower level of dyspnoea at discharge (1.9±0.3 vs. 2.5±0.2 points in those admitted directly to the general ward, *p* = 0.02), although they presented a similar degree of dyspnoea at admission (7.5±0.2 vs. 7.5±0.3 points, respectively, *p* = 0.98). The intensity of dyspnoea at hospital discharge was different according to COPD severity (2.4±0.1 points in mild; 2.5±0.2 points in moderate; 3.6±0.2 points in severe/very severe, *p*=0.02). Patients with preserved left ventricular ejection fraction had higher intensity of dyspnoea at discharge (2.7±0.2 points) compared to reduced left ventricular ejection fraction (2.0±0.3 points), *p*=0.03.

New York Heart Association functional class at hospital discharge was similar in the two groups (I or II: 142 [74.7%] in HF vs. 48 [71.6%] in RD, *p* = 0.62), and 67 (26.1%) remained at discharge in functional class III or IV (48 [ 25.3%] in HF and 19 [28.4%] in RD, *p* = 0.62).

Dyspnoea at discharge was higher in patients with depression than in those without (3.3±0.3 vs. 1.6±0.2 points, *p* < 0.0001), with no relevant differences regarding dyspnoea intensity at admission (7.7±0.2 vs. 7.3±0.2 points, respectively, *p* = 0.12), nor in the highest intensity during hospitalization (5.9±0.3 vs. 5.6±0.2 points, respectively, *p* = 0.33).

The independent predictors for severe dyspnoea at discharge by logistic regression modelling were age, female sex, depression, not admitted in an intensive care unit, and respiratory disease (Tables 3 and 4).

Age was also a predictor of higher degree of dyspnoea (as a continuous variable) at hospital discharge. Regarding the magnitude of dyspnoea decrease during hospital admission and its relation with treatment administered, the unique independent predictor of a larger decrease in dyspnoea during the course of hospitalisation was opioid treatment (*p*=0.028).

Data regarding the score of pain are also shown in Fig. 1. Patients with HF reported higher pain scores than those with RD, although the differences were not significant at discharge. A total of 31 patients (12.2%) presented with disabling pain at discharge. Scores on the different dimensions of EuroQoL 5d are described in Table 5. Compared with patients with RD, those with HF had more mobility problems and had lower scores in Visual Analogic Scale rating quality of life, and lower index values. They also presented more pain and felt more depressed, but these results did not reach statistical significance.

## Discussion

We found that patients admitted for HF and RD had an important reduction in health-related quality of life, in part as a result of dyspnoea. Dyspnoea is severe in the majority of patients at admission and improves during hospitalization, but remains intense at hospital discharge in about a quarter of patients. Opioids were seldom prescribed. Interestingly, HF patients had less dyspnoea than patients with RD but, despite that, they presented worse quality of life.

The higher burden of dyspnoea among patients admitted for RD could be expected, as HF patients present this symptom mainly in relation with pulmonary congestion. In fact, in those suffering from right-sided HF, dyspnoea is less notable and peripheral congestion prevails. Few studies have assessed the progress of dyspnoea during hospital admission. Dinino et al. [24] showed that dyspnoea severity improved rapidly during hospitalization. These authors found higher dyspnoea at baseline and admission and slower resolution of dyspnoea symptoms in patients with RD with respect to HF. Our results are consistent with this previous experience.

We also found that female sex was an independent predictor of severe dyspnoea at discharge. Previous studies have shown that women are a particularly vulnerable group [25, 26], with an increased risk of depressive symptoms added to breathlessness [1]. In fact, although many patients were anxious or depressed, treatment with antidepressants was administered to a low proportion of patients (13.2%). Depression was probably underdiagnosed too, as it can be masked by other symptoms common in HF and RD [27, 28], and diagnosis was performed using a questionnaire mainly designed to quantify dyspnoea. The benefits derived from depression treatment are substantial, as it improves morbidity and mortality [29] and antidepressants are safe in patients with HF and RD [30]. It is difficult to establish if dyspnoea caused depression or if low mood was responsible, in part, for the sensation of shortness of breath. In any case it seems to be room for improvement in its management.

Despite the frequency of severe dyspnoea and the fact that there are effective tools for treatment, such as opioids [31, 32], an extended therapeutic nihilism in patients with persistent dyspnoea has been described [33]. In our study only 27% of patients with severe dyspnoea at discharge received a medical action addressed to improve it. This is a two-way phenomenon, as 23% used no method for dyspnoea relief before admission and 41% had no previous communication with their general practitioner regarding dyspnoea.

There is a widespread non-familiarity with the use of scores to quantify the intensity of dyspnoea [34]. Our data emphasize the importance of quantifying dyspnoea and to have a proactive attitude in its management. In this regard, the first step should be to improve the professional skills to recognize, quantify, and treat dyspnoea. A previous experience in primary care with COPD has shown positive outcomes [35]. Even a small difference in the degree of dyspnoea is important and 1 point of perceived improvement in the numerical scale can be a great relief for the patient [6]. In spite of this data, we found that opioid use was unusual and only 5% received continuous treatment.

Interestingly, admission to an intensive care unit was a protective factor for dyspnoea at discharge, even though dyspnoea at admission was similar to patients admitted directly to the general ward. The justification of this effect is not clear, but is probably related with the twice-higher rate of opioids prescription in intensive care with respect to general ward.

Although patients suffering from HF had less dyspnoea at discharge than those with RD, they had a worse quality of life, mainly due to mobility limitations. Mobility problems are more common in HF and have a multifactorial origin, including skeletal muscle anomalies due to reduced blood supply, also called HF-related myopathy [36]. Pain was also more frequent in HF patients compared to the ones with RD. This could be, in part, explained by the occurrence of angina, as almost half of our HF patients had ischemic heart disease.

Our study supports the need for improvement in the caregiving to patients with HF and RD, in whom dyspnoea is a disabling symptom that worsens quality of life and surpasses the importance of other symptoms, like pain.

## Limitations

This is a cross-sectional study in a hospital setting, with no after-discharge follow-up. Also, due to its monocentric nature, its generalizability may be limited and our data might not represent daily practice and reality of other hospitals. In order to facilitate information collection and minimize data loss we administered a single survey on the day of discharge, so patients may have overstated or minimized the intensity of dyspnoea at hospital admission. This design had a more focused approach with daily practice. We included all patients admitted with HF, so the prevalence of dyspnoea may have been underestimated, due to the presence of strictly right-sided HF in some patients. Finally, most of our patients presented HF so there may have been a lack of statistical power to find significant differences between those patients and the low number of patients RD in some variables.

## Conclusions

About a quarter of patients admitted with HF or RD persist with severe dyspnoea at discharge. Opioids are probably underused. HF patients have less dyspnoea than patients with RD but, despite this fact, present worse quality of life.

## Additional file

Additional file 1: Appendix 1: Survey administered the day of hospital discharge to patients admitted for heart failure, COPD or pulmonary fibrosis. Patient’s questionnaire administered the day of hospital discharge to quantify quality of life, dyspnea assessment and relief methods. (DOCX 106 kb)

## Abbreviations

COPD: Chronic obstructive pulmonary disease
HF: Heart failure
MEDD: Morphine equivalent daily dose
NRS: Numerical rating scale
RD: Respiratory disease
