# Supplementary material for: Degree of dyspnoea at admission and discharge in patients with heart failure and respiratory diseases
Source: BMC Palliat Care. 2017 May 22;16:35. doi: 10.1186/s12904-017-0208-x (PMC5441077; doi:10.1186/s12904-017-0208-x)
Supplement: Additional file 1: — Appendix 1: Survey administered the day of hospital discharge to patients admitted for heart failure, COPD or pulmonary fibrosis. Patient’s questionnaire administered the day of hospital discharge to quantify quality of life, dyspnea assessment and relief methods. (DOCX 106 kb) [file 12904_2017_208_MOESM1_ESM.docx]

**Appendix 1.** Survey administered the day of hospital discharge to patients admitted for heart failure, COPD or pulmonary fibrosis.

Patient’s number: _________________________________

Date of inclusion: ____/____/____

**Who do you live with?** (Mark the best option)

🞎Alone

🞎With my family

🞎In a residency

**Which methods do you use when you feel shortness or breath or fatigue?** (You can mark more than one option)

🞎Oxygen

🞎Continuous Positive Airway Pressure (CPAP)

🞎Inhalers

🞎Fan

🞎Humidifier

🞎Sleeping with high headboard or more than one pillow

**Have you ever told your Family Doctor about your fatigue or shortness of breath?**

🞎 No 🞎 Yes

**Do you think that the treatment you have received could be better?**

🞎 No 🞎 Yes

**Has your fatigue or shortness of breath improved during admission?**

🞎 No 🞎 Sí

**Did you call the doctor or ask for help because you felt fatigue or shortness of breath?**

🞎 No 🞎 Yes

**Mark the option that best meets how you feel now.**

🞎 I do not feel any physical activity limitation due to fatigue.

🞎 I feel a slight limitation because of my breathlessness

🞎 Great limitation of my physical activity because of my breathlessness

🞎 I feel shortness of breath at rest, and I feel limitations to do any activity

**Degree of breathlessness from 0 to 10 (0 no breathlessness and 10 feeling like drowning in the sea)**

Before admission🞎 Maximun during admission 🞎 Right now🞎

**Degree of pain from 0 to 10 (0 no pain, 10 the most severe pain ever felt)**

Before admission🞎 Maximun during admission 🞎 Right now🞎

**Appendix 2.** The EuroQol 5 d is an instrument for measuring the quality of life related to health. The patient values his health in severity levels in five dimensions (mobility, personal care, daily activities, pain, anxiety) and also on a visual analog scale. Each of the 5 dimensions has 3 levels of potential severity: no problems, some problems or moderate problems and severe problems. The analog scale assessment consists of a millimeter line from 0 (worst imaginable health state) to 100 (best imaginable health state). A third element is the global index values obtained for each health status generated by the instrument, which can range from the value 1 (the better health posible) and 0 (death). There are also negative values that are rated as worse than death. (Appendix 2).^25-27^

**EuroQol 5d survey**. EuroQol questionnaire was also included in the survey administered to patients the day of hospital discharge.

Under each heading, please tick the ONE box that best describes your health TODAY.

**Mobility:**

- I have no problems in walking about 🞎
- I have some problems in walking about 🞎
- I am confined to bed 🞎

**Self-care**

- I have no problems with self-care 🞎
- I have some problems washing or dressing myself 🞎
- I am unable to wash or dress myself 🞎

**Usual activities (e.g. work, study, housework, family or leisure activities*)***

- I have no problems with performing my usual activities 🞎
- I have some problems with performing my usual activities 🞎
- I am unable to perform my usual activities 🞎

**Pain/discomfort**

- I have no pain or discomfort 🞎
- I have moderate pain or discomfort 🞎
- I have extreme pain or discomfort 🞎

**Anxiety/Depression**

- I am not anxious or depressed 🞎
- I am moderately anxious or depressed 🞎
- I am extremely anxious or depressed 🞎

To help people say how good or bad a health state is, we have drawn a scale (rather like a thermometer) on which the best state you can imagine is marked 100 and the worst state you can imagine is marked 0.

We would like you to indicate on this scale how good or bad your own health is today, in your opinion. Please do this by drawing a line from the box below to whichever point on the scale indicates how good or bad your health state is today.

*The best health you can imagine*


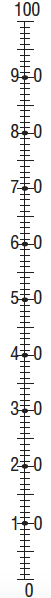


Your health today = 🞎

*The worst health you can imagine*
